# Factors Associated with Driving Ability and Changes After Immobilization of the Right Lower Limb: A Driving Simulator Study

**DOI:** 10.3390/jcm14041396

**Published:** 2025-02-19

**Authors:** Young Cheol Kim, Moo Sik Lee, Byung Hak Oh, Youn Moo Heo, Tae Gyun Kim, Se Jong Yoo, Hyun Jin Yoo

**Affiliations:** 1 Department of Orthopedic Surgery, Konyang University College of Medicine, Daejeon 35365, Republic of Korea; cast9125@hanmail.net (Y.C.K.); sebslab@hanmail.net (B.H.O.); hurym1973@hanmail.net (Y.M.H.); ktk1113@hanmail.net (T.G.K.); 2 Department of Preventive Medicine, Konyang University College of Medicine, Daejeon 35365, Republic of Korea; mslee@konyang.ac.kr; 3 Department of Radiological Science, Konyang University, Daejeon 35365, Republic of Korea

**Keywords:** driving ability, right lower limb, orthopedic immobilization, responses recorded

## Abstract

**Introduction:** Patients with orthopedic injuries often require cast immobilization therapy using casts. Driving with a cast on the right lower limb restricts many things. This study aimed to investigate the factors associated with driving capacity after orthopedic fixation of the right lower limb in healthy adult volunteers. This study’s hypothesis was that the orthopedic splint immobilization would have delayed reaction when in a driving simulation. **Materials and Methods:** We carried out an experimental study between 17 April 2023 and 19 May 2023. We set up the study in two phases: a driving simulation experiment without immobilization with a cast on the right lower limb and a driving simulation experiment with immobilization with a cast on the right lower limb. The data collected through the questionnaire were then analyzed in R version 4.2.2. **Results:** A total of 47 individuals participated in the study with a mean height of 167.68, of which 68.09% were females. Overall, 78.7% of the study participants could not drive after immobilization with a cast on the right lower limb. There was a significant difference between participants who could drive and those who could not drive by height (*p* = 0.04), age (*p* = 0.038), and body type (*p* = 0.046). **Conclusions:** Our study demonstrates that an individual’s height, age, and body type are associated with an individual’s driving ability after orthopedic immobilization of the right lower limb. Our findings suggest that regulations regarding the ability to drive after orthopedic immobilization of the right lower limb must be reconsidered.

## 1. Introduction

Orthopedic injuries are common in daily life. Globally, the burden of orthopedic injuries is increasing and has been reported to cause significant economic burdens [1,2]. A study by P. Hemmann et al. [3] to explore the epidemiology of lower extremity injuries in adults found out that lower extremity fractures increased by 4.5% between 2002 and 2017. In the United States, the gross burden of orthopedic injury is substantial with reports of about 1.6 million emergency orthopedic surgeries in 2020 [4]. Among the damage to the lower extremities, 84.4% require a short leg splint, and the right side of them is researched to be about 50.8% [5]. Ankle sprains occur in 1/10,000 sports-related injuries, with 850,000 cases of lateral corner ankle injury occurring annually in the USA and a recurrence rate of 70% [6]. In addition, it is common in men in their 20s and 30s who are the most active and has a high recurrence rate and a high probability of sequelae of instability [7].

In South Korea, in the pre-COVID-19 period, the prevalence of adolescence orthopedic injuries was 3.10% whereas in the pandemic period, the prevalence decreased to 2.42% [8]. Despite a temporary decrease in the number of cases of orthopedic injuries as a result of restricted outdoor activities due to the COVID-19 pandemic in 2020, the number of lower limb fracture patients is projected to increase in the post-COVID-19 pandemic period [8,9,10]. In high-income countries, road traffic injuries result in a significant number of deaths in people 15–29 years old, with 75% of deaths occurring in men [11].

Patients with orthopedic injuries often require cast immobilization therapy using casts regardless of surgical intervention, which can last from weeks to months depending on individual recovery [2,3]. During the immobilization period, most individuals experience significant limitations, often leading to major inabilities, one of which is the inability to drive effectively [4,8,9]. The right lower limb is primarily responsible for controlling the accelerator and brake pedal, stability and support, and for handling simple and complex emergencies [12]. Driving with a cast on the right lower limb entails complex physical burdens including restricting the range of joint motion necessary for driving, impairing sensory function, and making fine manipulation difficult [4,9,11]. Also, muscle loss occurs rapidly after casting, limiting the proper use of force [13]. In addition to the physical burden, immobilization of the right lower limb has also been associated with significant mental burden [14].

A study by S. Lissek et al. [15] found that immobilization by cast wearing significantly affected tactile perception and also shrunk the somatosensory cortical map. A study to determine the effects of right lower limb orthopedic immobilization on braking function also concluded that wearing an immobilization device on the right lower limb lengthens the emergency braking time [16]. Prior to the study by A. Waton et al. [17], there had been few studies that had investigated the effects of knee restriction on braking performance. However, A. Waton et al. [17] in their study showed that there was a significantly longer braking time among participants who had a cast fixed on their right leg than the normal braking time. A similar study by D. Dammerer et al. [18] also determined that there was a significant impairment in brake response time among participants who had knee braces on their right leg. These physical and mental changes can pose risks while driving, especially during emergencies, resulting in brake and accelerator confusion, which could result in road fatalities.

## 2. Materials and Methods

### 2.1. Study Design

We carried out an experimental study to determine the factors associated with an individual’s car driving ability after applying a cast on the right lower limb between 17 April 2023 and 19 May 2023 in Daejeon, South Korea.

### 2.2. Study Participants

Individuals were eligible to participate in this study on condition that they were adults aged between 18 and 59 years. In addition, participants were to possess a valid driver’s license and use only the right foot for accelerating and braking. The exclusion criteria for this study were a history of drug or alcohol abuse; sleep disorders; musculoskeletal impairment; and/or motion sickness. Prior to the start of this study, all participants provided written informed consent before taking part in the study.

### 2.3. Experiment Setting

**Figure 1:**
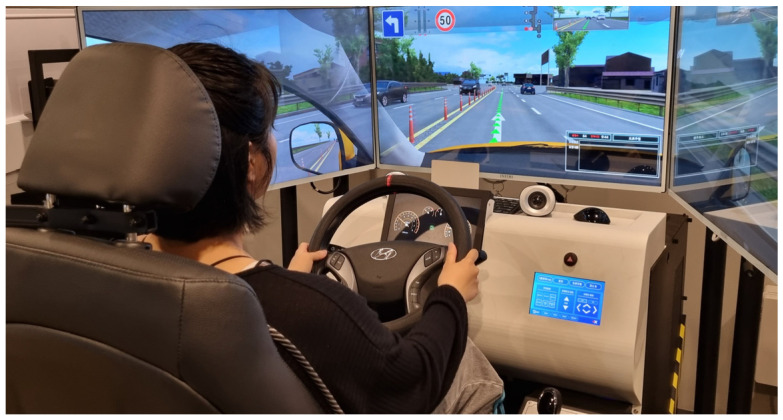
Indoor driving simulation facility.

After obtaining administrative authorization, the experiment was performed in an indoor driving simulation facility in a driving academy in Daejeon, South Korea (Figure 1). 

Together with a certified driving instructor, we facilitated the session for all participants. The first step of the experiment was familiarization with the driving simulator and instructions for the experiment. Participants were encouraged to sit and adjust the seat and steering wheel as they would normally do in their vehicles. The experiment included a test of driving ability, emergency braking episodes, driving on a straight-line track, and be able to maintain a speed of 50 km/h. This experiment was carried out in two phases.

**Figure 2:**
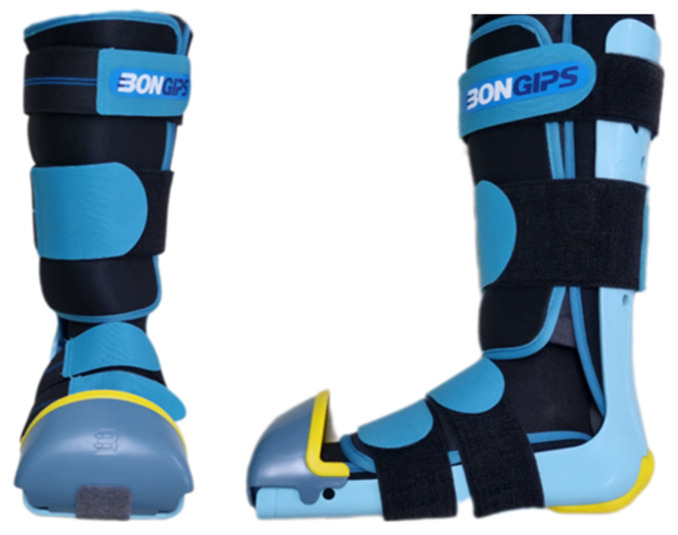
The immobilization device (Bongips).

In phase one, participants underwent the driving simulation experiment in their natural state without applying a cast on the right lower limb, after which they were administered a questionnaire by a trained research assistant. In phase two, each participant’s right lower limb was immobilized in a cast. Participants were required to walk a distance of 50 m to become familiar and comfortable with wearing the immobilization device (Figure 2), after which the driving simulator experiment was conducted, and a post-experiment questionnaire was administered.

All participants underwent the driving simulation experiment under the same conditions before and after applying the cast using indoor driving simulation equipment.

### 2.4. Survey Variables

In order to answer the study’s research questions, we developed a structured questionnaire. The survey questionnaire included sections on sociodemographic characteristics, information on when, method, and duration since acquisition of participant’s driver’s license, and physical and mental characteristics related to driving ability before and after immobilization with the cast. The questionnaire was pre-tested, and feedback received. Then, the questionnaire was revised and finalized based on the feedback.

The study participants’ sociodemographic variables were first collected and included age, gender, height, vision (left and right), occupation, level of education, body type, and health status. We classified the age into two categories (years old) ≤25 and >25; educational level was grouped into high school graduates (high school degree holders and below) and university/professional degree; occupation was grouped into student and employed. Body type was grouped into skinny, average, and overweight. Study participants were also asked to assess their health status, and it was classified into health free/disease and sick.

In order to assess the factors associated with driving ability after immobilization of the right lower limb in Korea, questions were developed based on a review of the relevant literature. Furthermore, after the practical session with the driving simulator in phases 1 and 2, participants were asked questions on various driving-related abilities, which included accelerator control, physical strain and pain, ability to deal with emergencies, occurrence of traffic collision, brake control/sudden braking, violation of traffic light, adherence to the stop line, safe left and right turns. The questionnaire was estimated to take 10 min on average for each participant at the end of each phase.

### 2.5. Statistical Analysis

Study participants’ responses from the survey were recorded in Excel, cleaned, and exported to a local installation of R version 4.2.2 for analysis [17]. We started the analysis of data by performing a descriptive summary for all variables. The numerical variables were summarized as a mean (±SD), and categorical variables were summarized by frequency and percentage. To evaluate the association between sociodemographic factors and the ability to drive after immobilization with a cast on the right lower limb, student *t*-test was used for numeric variables, (height, vision left, and right) and the Fisher’s exact test for categorical variables (age group, gender, occupation, education, body type, license acquisition, driving experience, and experience of immobilization on right lower limb). To evaluate changes in a participant’s driving-related characteristics before and after immobilization with a cast on the right lower limb, McNemar test was used. The level of statistical significance was set at a *p*-value < 0.05, and all statistical tests were two-tailed.

## 3. Results

A total of 47 individuals participated in the study with a mean height of 167.68, of which 68.09% were females. In this study, 63.83% of the participants were students and were aged 25 years and below (36.1%). Most participants acquired their driver’s license (68.09%) and had actual driving experience of 3 years and less (82.98%). Among the study participants, 38.30% had previous experience of cast immobilization with 19.15% of them reporting immobilization in the right lower leg (Table 1).

Overall, 78.7% of the study participants had no ability to drive after immobilization with a cast on the right lower limb while 21.3% had driving ability after immobilization. There was a significant difference between participants who had the ability to drive and those who did not have the ability to drive by height (*p* = 0.04), age (*p* = 0.038), and body type (*p* = 0.046) (Table 2).

Furthermore, we examined changes in driving-related characteristics before and after immobilization with a cast on the right lower limb. We observed significant changes in the participants’ ability to brake (*p* < 0.001) and accelerate (*p* < 0.001). There was also a significant difference in changes in compliance with road surface markings (*p* < 0.001), traffic signals (*p* < 0.001), experiment stop lines (*p* < 0.001), and safe left and right turns (*p* < 0.001). After immobilization with a cast on the right lower limb, 91.49% of the study participants reported a mental burden while 61.7% reported experiencing physical pain and/or discomfort (Table 3).

After the experiment, 93.62% of the study participants reported no intention to drive after immobilization with a cast on the right lower limb and all participants reported increased awareness of incompetent driving and violation of safety obligations after immobilization with a cast on the right lower limb. In addition, participants recommended the need for temporary driving restriction regulations (93.62%), punishment/fines (82.98%), and new technological methods (%) that may facilitate driving for individuals requiring immobilization with a cast on the right lower limb.

## 4. Discussion

Globally, orthopedic injuries occur regularly and most often need immobilization as part of the healing process [19,20,21]. This immobilization usually hinders individuals’ daily activities including driving. Although many studies have been carried out on different aspects of orthopedic immobilization of the limb, we found no studies on the factors associated with driving ability after orthopedic immobilization of the right lower limb in Korea [15,16,17]. The objective of this study was to determine the factors associated with driving ability after orthopedic immobilization of the right lower limb.

We observed that driving ability was associated with height, age, and body type. Previous studies have shown that taller individuals have better visibility and reach, potentially leading to improved control and awareness on the road [22]. Also, the empirical literature demonstrates that the relationship between an individual’s age and driving ability is complex and multifaceted. While younger drivers may possess quicker reflexes and better physical capabilities, older drivers often have more experience and cautious driving habits [23,24,25,26]. In our study, we found that body type was statistically significant with driving ability. Several studies suggest that overweight does not necessarily mean fatty [27]. Furthermore, in the case of overweight, which is caused by more muscles, driving ability can be interpreted as being more dominant even in the immobilization state due to functional superiority [28].

In addition, our studies showed significant changes in abilities to control braking and accelerating before and after orthopedic immobilization of the right lower limb. This was similar to reports by J. C. Murray et al. [16] who reported that orthopedic immobilization of the right lower limb impairs the driver’s ability to brake and accelerate. A multitude of factors have also been known to affect a driver’s ability to brake or accelerate including age, sex, expectancy when faced with danger, urgency of the situation, and cognitive load on the driver [29]. In addition, in this study, we observed violations of road surface markings, traffic signals, and noncompliance with experiment stop lines and turns after orthopedic immobilization of the right lower limb. Although a driver’s ability to respect road surface markings and traffic signals and to rapidly bring their vehicle to a stop in situations of emergency is an important characteristic for safe driving, after orthopedic immobilization of the right limb, these functions are impaired [30,31].

With the development of medicine and surgery, the importance of early rehabilitation is emphasized in many areas of orthopedic [32,33]. Nevertheless, the prognosis of immobilization still does not decline in the lower extremities compared to brace and surgical treatment [21,34,35]. In addition, there were no differences between the prognosis after rapid rehabilitation after surgery and recovery after immobilization for a certain period [32,36,37]. Some studies have also confirmed that early immobilization’s prognosis is superior to rapid rehabilitation and assistance in ankle damage that does not require surgery [21,38,39]. Therefore, immobilization is an inevitable treatment to some extent, especially in ankle joint damage. Early exercise is also needed for muscle strength and muscle activity after surgery [34,36,37]. In addition, immobilization has a bad effect on the regression of joint cartilage and synovial activity [7]. Therefore, only an appropriate period of immobilization according to the injured site and degree will be needed.

The major strength of our study was the use of the driving simulator that provided a controlled and safe environment to assess the impact of immobilization of the right lower limb on driving ability. However, our study had some limitations. In this study, none of the participants had lower right limb injuries and, consequently, had no pain, or other symptoms that occurred due to pain (such as lack of sleep); in addition, there was no consideration for painkillers and anti-spasmodic medications that could affect driving ability. We recruited randomized study participants, but there was an imbalance in the gender. The association with gender was not statistically significant in the results, but it can be a limitation by affecting other variables (height and weight). Our study supports and adds to previous work suggesting that immobilization of the right lower limb changes the ability to control braking and accelerating, keeping to road surface markings and traffic signals, and noncompliance with experiment stop lines and turns.

In addition, the sample size was limited, and the participants were relatively young and had no comorbidities. Consequently, the results cannot be readily applied to patients with orthopedic injuries. However, we consider that our study represents substantial progress in this field of research, as it provides a glimpse into the factors and the changes associated with immobilization of the right lower limb and driving ability.

## 5. Conclusions

Our study demonstrates that an individual’s height, age, and skin type are associated with an individual’s driving ability after orthopedic immobilization of the right lower limb. Our findings suggest that regulations regarding the ability to drive after orthopedic immobilization of the right lower limb must be reconsidered.

## Figures and Tables

**Table 1:** Sociodemographic characteristics of study participants (N = 47).

Variables	Mean ± SD
Height	167.68 ± 8.38
Vision left	1.004 ± 0.303
Vision right	0.97 ± 0.31
	N (%)
Age group	
≤25	36 (76.6)
>25	11 (23.4)
Gender	
Female	32 (68.1)
Male	15 (31.9)
Occupation	
Student	30 (63.8)
Employed	17 (36.2)
Education level	
High school	25 (53.2)
More than	22 (46.8)
Body type	
Average	30 (63.8)
Overweight	11 (23.4)
Skinny	6 (12.8)
License acquisition	
≤3 years	32 (68.1)
>3 years	15 (31.9)
Driving experience	
≤3 years	39 (83.0)
>3 years	8 (17.0)
Experience of immobilization on right lower limb	
Yes	9 (19.2)
No	38 (80.8)

SD: Standard deviation, N: number.

**Table 2:** Bivariate associations between sociodemographic factors and the ability to drive after wearing a cast on the right lower limb.

Variables	Driving Ability After Immobilization		*p*
	Yes	No	
Height	172.30 ± 9.59	166.43 ± 7.69	0.04
Vision left	1.05 ± 0.40	0.99 ± 0.28	0.82
Vision right	1.07 ± 0.37	0.95 ± 0.29	0.54
	N (%)		
Age group			0.038
≤25	10 (27.8)	26 (72.2)	
>25	0 (0.0)	11 (100.0)	
Gender			0.704
Female	6 (18.8)	26 (81.2)	
Male	4 (26.7)	11 (73.3)	
Occupation			0.07
Student	9 (30.0)	21 (70.0)	
Employed	1 (5.9)	16 (94.1)	
Education			0.73
High school	6 (24.0)	19 (76.0)	
More than	4 (18.2)	18 (81.8)	
Body type			0.046
Average	4 (13.3)	26 (86.7)	
Overweight	5 (45.5)	6 (54.6)	
Skinny	1 (16.7)	5 (83.3)	
License acquisition			0.46
≤3 years	8 (25.0)	24 (75.0)	
>3 years	2 (13.3)	13 (86.7)	
Driving experience			0.17
≤3 years	10 (25.6)	29 (74.4)	
>3 years	0 (0.0)	8 (100.0)	
Experience of immobilization on right lower limb			1
Yes	2 (4.3)	7 (14.9)	
No	8 (17.0)	30 (63.8)	

*p* was estimated using the student *t*-test for numeric variables and Fisher’s exact test for categorical variables.

**Table 3:** Changes in participant’s driving-related characteristics before and after immobilization with a cast on the right lower limb.

Variables	Before		Immobilization		*p*
	Yes	No	Yes	No	
Brake control issues	9 (19.15)	38 (80.85)	35 (74.47)	5 (10.64)	<0.001
Accelerator control issues	6 (12.77)	41 (87.23)	34 (72.34)	6 (12.77)	<0.001
Violation of road surface markings	5 (10.64)	42 (89.36)	26 (55.32)	14 (29.79)	<0.001
Traffic signal violation	4 (8.51)	43 (91.49)	26 (55.32)	14 (29.79)	<0.001
Compliance with experiment stop lines	43 (91.49)	4 (8.51)	19 (40.43)	21 (44.68)	0.001
Safe left and right turns	45 (95.74)	2 (4.26)	16 (34.04)	24 (51.06)	<0.001
Mental burden	N/A	N/A	43 (91.49)	4 (8.51)	
Physical pain and/or discomfort	N/A	N/A	29 (61.70)	18 (38.30)	

*p* was estimated through Mc Nemar test.

## Data Availability

The data presented in this study are available on request from the corresponding author. The data are not publicly available due to privacy.
